# Filling Small Round Cavities with Gold

**Published:** 1904-06

**Authors:** R. E. Sparks

**Affiliations:** Kingston, Can.


### FILLING SMALL ROUND CAVITIES WITH GOLD.

By R. E. Sparks, Kingston, Can.

Take a third sheet of No. 5 non-cohesive gold foil, folded into a very narrow ribbon, and insert one end into the cavity. With a hand-pressure plugger catch the ribbon far enough from the end just introduced to form a fold long enough to reach to the bottom of the cavity, while the loop of the fold extends slightly beyond the orifice. Continue this process until the cavity is a little more than full. Then condense and finish as in any ordinary case. If the cavity be extraordinarily deep for its size, fill to, say, one-half its depth and condense. Use this as the floor of the cavity, and proceed as above described. Non-cohesive gold spreads upon pressure, and accommodates itself to the size and shape of the cavity without balling, as is the case with cohesive. Besides, such a cavity may be filled in much less time with the former than with the latter.—*Dominion.*

